# Associations among executive function, cardiorespiratory fitness, and brain network properties in older adults

**DOI:** 10.1038/srep40107

**Published:** 2017-01-05

**Authors:** Toshikazu Kawagoe, Keiichi Onoda, Shuhei Yamaguchi

**Affiliations:** 1 Department of Neurology, Faculty of Medicine, Shimane University, 89-1, Enya-cho, Izumo city, Shimane, 693-8501, Japan

## Abstract

Aging is associated with deterioration in a number of cognitive functions. Previous reports have demonstrated the beneficial effect of physical fitness on cognitive function, especially executive function (EF). The graph theoretical approach models the brain as a complex network represented graphically as nodes and edges. We analyzed several measures of EF, an index of physical fitness, and resting-state functional magnetic resonance imaging data from healthy older volunteers to elucidate the associations among EF, cardiorespiratory fitness, and brain network properties. The topological neural properties were significantly related to the level of EF and/or physical fitness. Global efficiency, which represents how well the whole brain is integrated, was positively related, whereas local efficiency, which represents how well the brain is functionally segregated, was negatively related, to the level of EF and fitness. The associations among EF, physical fitness and topological resting-state functional network property appear related to compensation and dedifferentiation in older age. A mediation analysis showed that high-fit older adults gain higher global efficiency of the brain at the expense of lower local efficiency. The results suggest that physical fitness may be beneficial in maintaining EF in healthy aging by enhancing the efficiency of the global brain network.

The aging process is accompanied by a decline in numerous cognitive domains^1^,^2^. Physical exercise has been shown to be beneficial in protecting older adults from this cognitive decline, both in normal and pathological aging^3^,^4^,^5^,^6^. Regular physical activity has been recognized as the best among several strategies (e.g., intellectual activity, social participation, a healthy diet) for promoting general health, including physical as well as cognitive functioning^7^. Of the broad range of cognitive functions, executive function (EF) has been shown to have the greatest association with physical function^6^,^8^,^9^,^10^, and the effect of exercise on EF has been demonstrated experimentally^11^,^12^,^13^. Studies have also indicated that cardiorespiratory fitness results in greater positive effect on cognitive function than many other types of exercise^14^,^15^,^16^. Similar significant associations between cardiovascular fitness and cognitive function, especially EF, have been found^17^, despite some counterevidence^18^.

Several mechanisms underlying the exercise effect were originally suggested by animal studies and later supported in humans; these include neurogenesis, synaptic plasticity, neurotrophins, and cerebral blood flow^19^. Research on the effects of exercise on older adults has primarily focused on structural and functional changes of the brain in relation to cognitive improvement^20^. Erickson, *et al*.^21^ demonstrated that training in aerobic exercise increased spatial memory and hippocampal volume in older adults, reversing age-related losses in 1 to 2 years. The association between aerobic fitness level and EF was found to be mediated by the volume of gray matter in the frontal cortex^22^, and changes in the brain activation of older adults with aerobic exercise intervention have been accompanied by improvement in cognitive function^11^,^23^. Recently, the relationship between aerobic fitness level and level of EF was found to be mediated by frontal lateralization (i.e., compensatory neural recruitment) in older adults^24^ in accordance with the hemispheric asymmetry reduction in older adults (HAROLD) model^25^,^26^. Dupuy, *et al*.^27^ found significant positive associations among physical fitness, frontal brain oxygenation, and EF, suggesting, in accordance with previous studies, that a higher level of cardiorespiratory fitness is related to higher task-evoked hemodynamic responses in frontal areas. Evidence of an association between physical exercise and cognitive function is important because age-related cognitive decline can progress into neurodegenerative diseases, such as Alzheimer’s disease, which cause morbidity, loss of independence, and mortality^28^. In sum, prior studies have provided ample evidence that physical fitness improves EF by modulating its neural basis.

The technique of resting-state functional Magnetic Resonance Imaging (rsfMRI) is currently used to measure spontaneous neural activity and to evaluate regional brain fluctuation that occurs during rest or when a participant is not performing an explicit task or during rest. The rationale of this approach is that the brain displays persistent, spontaneous functional activity during rest^29^,^30^,^31^. This coherent spontaneous brain activity, or resting-state functional connectivity (rsFC), provide a means to assess the functional integrity of a broad range of brain networks in a relatively short period of time without focusing on a specific task-based processing system. Previous studies have shown that rsFC is important for healthy brain functioning^32^. Using rsfMRI, researchers have demonstrated that substantial changes of rsFC occur in normal and pathological aging^33^,^34^. A positive association between cardiorespiratory fitness and rsFC was recently demonstrated in several cortical networks of older adults^35^.

Another method of identifying biologically plausible brain networks is to model their topological organization using a graph theory based model. This approach graphically represents the brain as a complex network of nodes and edges. The basic conception has been well described in previous reviews^36^,^37^,^38^; in brief, the human brain is represented as a single integrative complex network, in which all brain regions and subnetworks are linked together into one complex system. Examining the overall network can provide new insight on how the brain is organized. This approach investigates the topological properties of the complex network of the brain to uncover the local and global organization of functional brain networks. As explained below, overall and regional “efficiency” can be calculated. Although some have questioned the validity of this technique^31^, when it has been applied to rsfMRI data, the network topology within an individual has been shown to be stable across time^39^,^40^. This approach has been used to demonstrate an effect of aging on properties of the cortical anatomical network^41^,^42^; however, no study has yet investigated the graph properties of a functional brain network from the perspective of the association between physical fitness and cognitive function in the aging population.

In this study, we attempted to replicate previous findings in this field (i.e., the association of EF and physical fitness) and to provide topological evidence about the organizational efficiency of the brain network of older adults. Based on previous studies of young adults and children^43^,^44^,^45^, there should be an association between overall network properties and cognitive and/or physical fitness in older adults. We also asked whether or not the network efficiency of specific local brain areas is associated with EF and physical fitness. Moreover, we attempted to discover the specific region(s) associated in common with EF and fitness. The research consensus indicates that the frontal areas play an important role in cognitive functioning^26^,^46^,^47^,^48^ and motor performance in aging^5^,^49^,^50^; however, other regions, including the subcortical areas, also appear to be important for older adults’ physical performance^51^,^52^,^53^,^54^. In sum, we examined the association between EF and physical fitness and its relationship with topological property in older adults. To the best of our knowledge, this is the first study to examine the relationship of brain network properties to cognitive and physical fitness. Common effective region(s), if these exist, may provide a clue to uncover a mechanism of cognitive and physical connections in old age.

## Result

### Behavioral data

**Figure 1:**
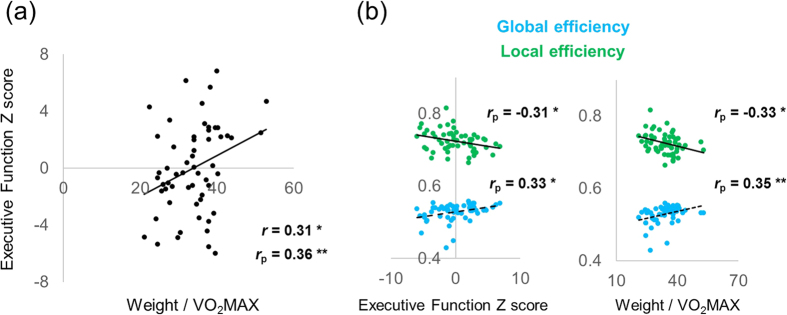
(**a**) Correlation between scores of integrated executive functions and VO_2_MAX. Coefficients of simple and partial correlations, excluding effects of age, sex, and years of education, are displayed. (**b**). Correlation of executive and physical indices with graph theoretical network properties. Only partial correlations are shown. Asterisks indicate p-values: *p < 0.05,**p < 0.01.

The demographic and behavioral data of participants are listed in Table 1. The correlation of integrated z-scores of EF (EF-Z) and VO_2_MAX is plotted in Fig. 1(a). The association was significant (*r*_p_ = 0.36, *p* = 0.007), in accordance with previous studies, even with the consideration of confounding factors such as age, sex, and years of education. Behavioral data fit well with Japanese healthy age-matched normative data in MMSE^55^, FAB^56^, VFT, and WCST^57^. Unsurprisingly, EF-Z was significantly correlated with each separate index of EF (VFT: 0.61, FAB: 0.64, WCST (CA): 0.58, WCST (RT): −0.56, and KHT: 0.70; p < 0.001).

### Graph theory analyses

We computed global efficiency, local efficiency, and betweenness centrality as indices of network integrity that characterize how well information is communicated within the brain (see the Method section for more detail). To investigate first whether efficiency of the whole network was significantly related to EF and physical function, overall indices were calculated via correlation analyses, with age, sex, and years of education considered as nuisance factors. The results are shown in Fig. 1(b). The global efficiency of the whole brain network was positively and significantly correlated with EF-Z (*r*_p_ = 0.33, *p* = 0.011), and VO_2_MAX (*r*_p_ = 0.35, *p* = 0.008). The local efficiency of the whole brain network was also significantly correlated with EF-Z (*r*_p_ = −0.31, *p* = 0.018) and VO_2_MAX (*r*_p_ = −0.33, *p* = 0.013). Surprisingly, local efficiency was negatively associated with EF and fitness. As predicted, efficiency of the whole brain network in association with EF and physical fitness showed more economical connectivity in high-fit and high-EF older adults; however, local “subnetworks” had a decreasing presence. For betweenness centrality, the whole network property was not significantly correlated with either index (EF-Z: *r* = −0.03; VO_2_MAX: *r* = −0.04). Because of this null correlation, the ensuing analyses were conducted only for global and local efficiency. Furthermore, although aging is always associated with decreased gray matter volume, the above associations remained significant after controlling for gray matter volume, both for global efficiency (EF-Z: *r*_p_ = −0.29, *p* = 0.029; VO_2_MAX: *r*_p_ = −0.31, *p* = 0.020) and for local efficiency (EF-Z: *r*_p_ = −0.30, *p* = 0.027; VO_2_MAX: *r*_p_ = −0.31, *p* = 0.019). For the spatial network property, no region met the conservative threshold (FDR corrected, *p* < 0.05).

### Mediation analysis

**Figure 2:**
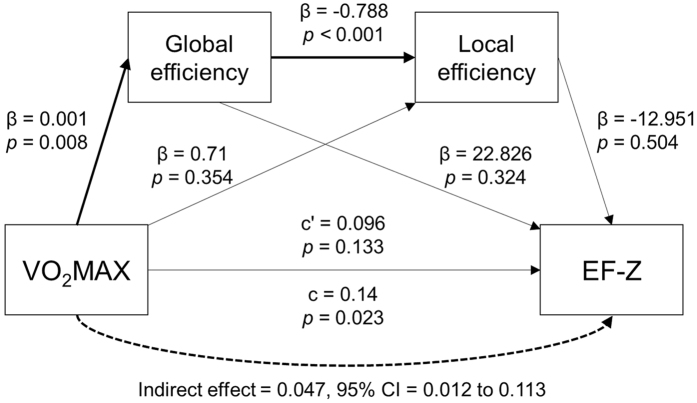
Mediation model of the relationship between physical fitness and executive function. β values indicate the coefficient of the arrow’s partial effect. c’ (c-prime) represents the direct effect and c represents the total effect of VO_2_MAX on EF-Z. Indirect effect coefficients and 95% bootstrap confidence interval indicate that the two mediators significantly mediated the relationship of VO_2_MAX and EF-Z. Bold arrows indicate significant effects.

A secondary mediation analysis was conducted to investigate whether brain topological properties mediated the relationship between VO_2_MAX and EF. The model was guided by the hypothesis that physical fitness can modify brain structure and function, as suggested by prior studies^6^,^9^,^12^,^21^. This model examined if global and local efficiency mediated the relationship of the independent variable, VO_2_MAX, and the dependent variable, EF-Z. The results indicated that the total model significantly fit the data (*F*(1, 55) = 5.71, *p* = 0.020, R^2^ = 0.10). The topological property mediator significantly mediated the independent and dependent variables (indirect effect = 0.047, 95% bootstrap confidence interval 0.012–0.113). The results are depicted in Fig. 2. Of course, reversing the disposition of global efficiency and local efficiency led to the same result for the model’s goodness of fit.

## Discussion

A substantial amount of research has indicated that there is an association between EF and many physical functions, especially physical fitness, in older adults^5^,^6^,^19^. First, we attempted to replicate the accomplished fact of a cognitive-physical association. An EF index was created from performance on several executive and attentional tasks^58^,^59^, and physical fitness was indexed by oxygen consumption, which is a gold standard of physical fitness^14^,^27^. The association was confirmed by simple and partial (considering age, sex, and years of education) correlation analyses, in accordance with previous studies^3^,^13^,^14^. In the present results, individuals with higher VO_2_MAX performed better in a series of executive function tests. Second, we found both the level of EF and physical function to be significantly correlated with rsFC as indexed by network properties, global efficiency and local efficiency. Finally, mediation analysis indicated that global and local efficiency mediated the relationship between physical fitness and EF.

The functional brain network property was calculated by a graph theoretical analysis in which the brain was regarded as an integrated complex network. Global efficiency indicates how efficiently information is integrated across the entire network, while local efficiency indicates how efficiently information is integrated between the immediate neighbors of a given network node, representing functional segregation. Betweenness centrality indicates the relative importance of a node within the overall architecture of the network. If the network is efficiently integrated, better performance should result. For example, van den Heuvel, *et al*.^32^ showed that higher IQ scores are associated with greater global efficiency in functional brain networks. Further, the organization of rsFC changes as a function of age^42^. A recent study has indicated that graph properties of rsFC predict working memory performance in younger and older adults, but in a different manner^44^. Local efficiency negatively affected working memory performance in both younger and older adults, but the effect of global efficiency was positive in younger adults and negative in older adults. This observation is consistent with the present study with respect to the association between rsFC properties and EF; that is, a positive relationship between EF and global efficiency and a negative relationship between EF and local efficiency. The present study suggests that the rsFC data analyzed via a graph theoretic method can predict the level of EF in older adults. The association with global efficiency found here is supported by previous rsFC studies indicating a positive relationship between rsFC and EF^59^,^60^. Further, global efficiency was positively related to level of physical fitness. This result can also be instinctively understood. Higher fit older adults achieve better cognitive functioning, which should be regulated by the structural and functional states of the brain^20^,^24^,^27^, and this can be captured even in a resting state^35^. We demonstrated such an association in older adults in topological rsFC data, especially for global efficiency.

In contrast to global efficiency, the present results for local efficiency are more counterintuitive. We found local efficiency to be negatively correlated with both EF and physical fitness level. A network has to maintain higher local efficiency to communicate most efficiently, and this is determined in small-world networks^61^. Even considering the previous suggestion that localized, functional specialization cannot fully account for the brain function^62^, the negative correlation of local efficiency and EF and fitness is difficult to interpret. However, as described above, this pattern of results concurs with previous studies that showed a negative effect of local efficiency on working memory performance in older adults^44^. We can therefore interpret this result as follows. High functioning individuals who have higher levels of both EF and physical fitness should tend to have higher global efficiency at the cost of lower local efficiency (e.g., which are more similar to random networks). The loss of functional segregation is one of the representative qualities of older adults. This *dedifferentiation*^46^ is characterized by reduced selectivity in brain regions that originally act as the neural basis of a specific function, for example, face processing^63^. This phenomenon may reflect the loss of local efficiency. On the other hand, the frontal region plays a very important role in *compensation*, defined as an increase in brain activities (always compared to younger controls) that serves as a beneficial counteraction to age-related performance decline^5^,^25^,^47^,^48^. The present correlation of graph theoretic properties with EF and physical fitness may reflect the tendency of high functioning individuals to have connections across a broader range of brain areas at the expense of local segregation. If this is so, it is understandable why local efficiency is negatively related to measures of EF and fitness. Increasing global efficiency which is by means of connecting a broader range of brain areas may contribute to higher task performance and resulted in deceased local efficiency. Based on previous reports, the direction of effects may be from a higher level of physical fitness to higher global efficiency and lower local efficiency, and finally to better cognitive function.

To confirm the flow of association among physical fitness, global/local efficiency, and executive function, we conducted a mediation analysis. The model significantly fit with the data. Although the direct effect of VO_2_MAX on EF was not significant, the indirect effects of two mediators were significant, resulting in a significant total effect. This model revealed that VO_2_MAX significant affects the brain property of global efficiency as well as local efficiency. Because the two mediators’ direct effect on EF-Z was not significant, we cannot draw a firm conclusion about the validness of the model. However, because the model was significant, we can say that the association between physical fitness and EF was modulated or mediated by the brain’s topological network.

We found no spatial information indicating that specific regions are important for both EF and physical fitness in older adults. Previous studies have consistently shown the frontal region to be most important in the association between EF and physical function, including fitness^11^,^24^,^27^. Other data have suggested that the subcortical regions are also important in this association^52^,^53^,^54^. However, we have evidence for significant relationships among topological properties in the whole brain rsFC, EF, and physical fitness, but not for regional contributions. Analyzing data from a larger more heterogenous sample over a broader age range might discover important regional aspects of network plasticity that are directly linked to executive function and mediated by physical fitness. Another limitation of this study was the measurement for VO_2_MAX. We estimated those values based on participants’ maximal heart rate and resting heart rate, without more direct measurement via a gas analyzer, as in Fick principle calculations. However, this methodological issue does not seem crucial in the present results, because previous research has indicated the estimated index is valid^64^.

In this study, we found that high-fit older adults tended to have higher general efficiency and lower local efficiency as topological neural network properties, and these were related to level of EF. The pattern of topological efficiency may be related to compensation and dedifferentiation in older adults. Finally, we did not find a significant role for betweenness centrality, which is another important index of topological properties^62^. Future work should for clarify the importance of “hub” regions in the association between physical and cognitive functioning in old age.

## Methods

### Participants

The participants were selected from the health examination system database at the Shimane Institute of Health Science. This database is a collection of medical, neurological, neuropsychological, MRI, and blood test data for individuals who underwent rsfMRI scanning and neuropsychological testing from December 2012 to September 2015. All older individuals included in this database (60 to 80 years old) were Japanese and had been living independently in the community without any psychiatric treatment. Older participants were excluded if there was any suspicion of cognitive impairment or cerebral injuries or abnormalities, including severe atrophy, cerebral hemorrhage, previous cerebral infarction, aneurysm, severe hypoplasia, empty sella, any kind of cyst, perivascular space, or vessel malformation. Radiologists and other medical doctors provided these interpretations. Individuals having a medical history of cancer, heart disease, or severe decrease in vision or hearing were also excluded, as were individuals with any history of cerebral disease, stroke, psychosis, or Parkinsonism. Finally, we excluded individuals if their data included any missing values in the present analyses. Most individuals did not contribute physical fitness data because of injury or their schedule. After screening, 57 participants (24 women) were included in the sample (mean age 68.33, SD 5.62, range 60 to 79). The study was done in accordance with the Declaration of Helsinki (1975, as revised in 2008) and the regulations of the Japanese Ministry of Health, Labour and Welfare. Shimane University medical ethics committee approved this study and All study participants provided informed consent.

### Neuropsychological measures

All participants underwent a neuropsychological assessment in the Japanese language, which consisted of the Mini-Mental State Examination MMSE^65^; Verbal Fluency Test VFT^66^; Frontal Assessment Battery FAB^56^,^67^; Wisconsin Card Sorting Test administered on the computer WCST^68^,^69^; and the “Kana-hiroi” test KHT^70^. These tests tap higher-order cognitive function or EF. For the VFT, individuals were asked to generate names from a specified category (i.e., vegetable) in one minute; this is a popular procedure for assessing verbal fluency. For the WCST, scores considered were the number of categories achieved (CA) and required time (RT). In the KHT, participants picked out and circle five kana letters corresponding to A, I, U, E, and O (Japanese vowels) as they occurred in a story written in kana that the participants read silently. The number of letters correctly identified in 2 min was scored. The MMSE was used for rough screening of cognitive impairment, with a cutoff point of 24/25. The scores on multiple tests of EF were integrated into a single index to address the so-called “task impurity problem”^58^,^71^ that arises from the assessment of EF using a single measure. These scores were calculated by taking the individual average score across the EF-related tests (i.e., VFT, FAB, WCST, and KHT), converting these into Z-score values based upon the entire sample of participants^59^.

### Physical measures

The system used here included an index of oxygen uptake (VO_2_MAX). VO_2_MAX is the gold standard index of physical fitness and cardiorespiratory health^14^,^27^ and indicates how well the body takes in oxygen^72^. The highest rate of oxygen consumption during exhaustive exercise is expressed as oxygen capacity per kilogram of body weight over time (mL/kg/min). This index is adequate for the investigation of the association of physical fitness and cognition because cardiorespiratory health and cognition can be mediated by vascular mechanisms, such as cerebrovascular reserve^73^. We estimated VO_2_MAX via ergometer (BE-350, Fukuda Denshi) in 12 minutes of 3-step testing. At the first step, the maximum load was estimated based on the participants’ sex, age, and self-judged stamina (high, medium, and low). The initial load was set at 25% of maximum load (P1, corresponding to 25% of oxygen consumption), and participants exercised on the ergometer for 2 mins. The average pulse rate in the last 30 secs were measured to set the second load (P2, corresponding to 50% of oxygen consumption), under which participants worked out for another 4 mins. The third load (P3, corresponding to 60% of oxygen consumption) was again based on the average pulse rate in the last 30 secs. Participants underwent another 4 mins of exercise. The load and average pulse rate in all conditions were used to calculate a regression formula based on Astrad’s nomogram^74^, and a formula built into the apparatus produced the VO_2_MAX value.

### fMRI data acquisition and preprocessing

Imaging data were acquired using a Siemens AG 1.5 T scanner (Symphony). Twenty-seven slices (each 4.5 mm thick) with no gap parallel to the plane connecting the anterior and posterior commissures were measured using a T2*-weighted gradient-echo spiral pulse sequence, with repetition time 2000 ms, echo time 30 ms, flip angle 90°, interleaved order, matrix size 64 × 64, field of view 256 × 256 mm^2^, and isotropic spatial resolution 4 mm. All participants experienced a 5 min rsfMRI scan after we instructed them to remain awake with their eyes closed. After the functional scan, T1-weighted images of the entire brain were obtained (192 slices, repetition time 2170 ms, echo time 3.93 ms, inversion time 1100 ms, flip angle 15°, matrix size 256 × 256, filed of view 256 × 256 mm^2^, isotropic spatial resolution 1 mm). We used Statistical Parametric Mapping software (SPM12, http://www.fil.ion.ucl.ac.uk/spm) for spatial preprocessing. The first five volumes were deleted to account for signal stabilization. The remaining images were realigned to remove any artifacts from head movements, with 6 parameters for bulk head motion and an additional 6 parameters for translational displacement, and corrected for differences in image acquisition time between the slices. The realigned images were normalized to Montreal Neurological Institute (MNI) template standard space and resliced with a 3 × 3 × 3 mm voxel size. Spatial smoothing was then applied with the full width at half maximum (FWHM) equal to 8 mm. After spatial preprocessing, temporal preprocessing was conducted via the Functional Connectivity Toolbox (CONN, http://www.alfnie.com/software). Those data were band-pass filtered (0.01–0.08 Hz) to temporal smoothing, regressing out for nuisance covariates including head movement time series, white matter signals, and cerebral spinal flood signals.

### Graph theory-based connectivity analysis

The protocol for graph theoretical analysis of rsfMRI involved several steps^37^,^38^. Ninety regions of interest (ROIs) were defined based on Automated Anatomical Labeling AAL^75^, and the mean time course of each ROI was extracted. Pearson’s correlation coefficients between all pairs of ROIs time courses were then computed, resulting in a 90 × 90 correlation matrix that represented functional connectivity. To construct an undirected and unweighted graph network, the matrix was converted into a binary network using a density threshold equivalent to the ratio between the number of edges and all possible edges. The matrices were thresholded at different network densities (0.10–0.30, stepped by 0.01), and the graph theoretical parameters were averaged over the density range. Topological parameters were calculated for each ROI of the matrix. From a large number of network parameters in graph theory, we selected relatively well-known indices: global efficiency, local efficiency, and betweenness centrality^76^. These three indices represent three types of network parameters: functional integration, functional segregation, and centrality, respectively. They are computed for each ROI via CONN and the Brain Connectivity Toolbox (https://sites.google.com/site/bctnet/).

Global efficiency is an index of functional integration that is defined as the average inverse of the shortest path lengths between nodes^76^, computed as follows:


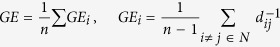


where *d*_*ij*_ is the shortest path length between nodes *i* and *j, n* is the number of nodes, and *GE*_*i*_ is the global efficiency of node I (i.e., nodal efficiency).

Local efficiency is a measure of functional segregation, which is the ability for specialized processing to occur within densely interconnected groups of brain regions, computed as follows:


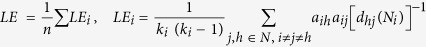


where *k*_i_ is the degree of node i, *a*_ih_ (*a*_ij_) is the binary connection status between nodes *i* and *h (j*), and *d*_hj_(*N*_*i*_) is the length of the shortest path between nodes *h* and *j* that contains only neighbors of node *i*. Higher local efficiency is interpreted as indicating the presence of functional subnetworks.

Betweenness centrality reflects the influence of the node over the information flow between all the pairs of other nodes in the whole network. Higher betweenness centrality indicates that the region functions as a hub. This index was calculated as:


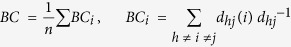


where *d*_hj_ denotes the number of shortest paths from *h* to *j (d*_jj_ = 1 by convention), and *d*_hj_(*i*) is the number of shortest paths from *h* to *j* that *i* lies on.

### Statistical analysis

Behavioral scores were analyzed by means of Pearson’s simple correlations and partial correlations. To investigate the relationship between graph theoretical property and EF and/or VO_2_MAX in older adults, we conducted partial correlation analyses while controlling for age, sex, and years of education. We examined the relationship between each graph property in the whole network and EF and/or VO_2_MAX. On this point, multiplicity irrelevant, so the statistical alpha was conventionally set at 0.05. An extended correlation analysis was conducted to test if the brain volume affected this relationship. To calculate brain volume, a MATLAB script written by Ged Ridgway was used (http://www0.cs.ucl.ac.uk/staff/g.ridgway/vbm/get_totals.m).

We conducted a secondary mediation analysis to examine the relationship of physical fitness and executive function. This was an hypothesis-driven model analysis focused on the role of mediator in the association of independent and dependent variables. To minimize the problems of modest sample size, bootstrapping procedures (2000 samples) were used to calculate indirect effects based on the coefficients of paths a and b (i.e., arrows adjacent to mediators in Fig. 2). This analysis was done via the PROCESS plugin for SPSS developed by Preacher and Hayes (http://processmacro.org/index.html). To determine significant effects, an alpha level of 0.05 was selected, and zero was not included in the 95% confidence interval. Those data were analyzed with SPSS 22.0 for Windows.

## Additional Information

**How to cite this article**: Kawagoe, T. *et al*. Associations among executive function, cardiorespiratory fitness, and brain network properties in older adults. *Sci. Rep.* **7**, 40107; doi: 10.1038/srep40107 (2017).

**Publisher's note:** Springer Nature remains neutral with regard to jurisdictional claims in published maps and institutional affiliations.

## Figures and Tables

**Table 1:** Demographic, neuropsychological, and physical fitness data [unit].

N = 57 (N of man: 33)	Mean	SD
Age [years]	68.3	5.6 (range: 60–79)
Years of education [years]	13.5	2.9
MMSE [point]	28.2	1.9
VFT [number]	15.9	3.7
FAB [point]	16.5	1.3
WCST (CA) [number]	4.7	1.0
WCST (RT) [sec]	599.6	139.2
KHT [number]	40.9	10.6
EF-Z [z-score]	6.38 × 10^−5^	3.1
VO_2_MAX [ml/kg/min]	33.8	6.6

CA: category achieved; RT: required time.
